# Of Time Gals and Mega Men: Empirical Findings on Gender Differences in Digital Game Genre Preferences and the Accuracy of Respective Gender Stereotypes

**DOI:** 10.3389/fpsyg.2021.657430

**Published:** 2021-05-10

**Authors:** Benjamin P. Lange, Peter Wühr, Sascha Schwarz

**Affiliations:** ^1^Department of Media Psychology, Julius-Maximilians-University of Wuerzburg, Wuerzburg, Germany; ^2^Department of Social Sciences, IU International University of Applied Sciences, Berlin, Germany; ^3^Department of Psychology, Technical University Dortmund, Dortmund, Germany; ^4^Department of Psychology, University of Wuppertal, Wuppertal, Germany

**Keywords:** digital game, genre, stereotype accuracy, gender differences, sex differences

## Abstract

We investigated the accuracy of gender stereotypes regarding digital game genre preferences. In Study 1, 484 female and male participants rated their preference for 17 game genres (gender differences). In Study 2, another sample of 226 participants rated the extent to which the same genres were presumably preferred by women or men (gender stereotypes). We then compared the results of both studies in order to determine the accuracy of the gender stereotypes. Study 1 revealed actual gender differences for most genres—mostly of moderate size. Study 2 revealed substantial gender stereotypes about genre preferences. When comparing the results from both studies, we found that gender stereotypes were accurate in direction for most genres. However, they were, to some degree, inaccurate in size: For most genres, gender stereotypes overestimated the actual gender difference with a moderate mean effect size.

## Introduction

Digital games, sometimes referred to as video games or computer games, are a popular medium and can even be considered a form of culture (Berger, 2017). Also, communication and media research has dedicated substantial research interest to digital games (e.g., Wulf and Baldwin, 2020). Moreover, the gaming industry has become a multi-billion-dollar business (Dillon, 2016). In 2020, the gaming market had reached a size of more than 100 billion USD. Within all digital media, the gaming industry is the biggest market (Statista, 2020). However, despite their pervasiveness, digital games are not played by everyone, nor are all players of digital games alike. Individual differences (e.g., with respect to gender) in digital game consumption are, thus, a worthwhile research subject (e.g., Cruea and Park, 2012), and so are peoples' beliefs (i.e., stereotypes) about individual differences in digital game preferences.

### Gender Differences in Digital Game Genre Preferences

Digital games are still widely considered so-called “boys' toys” or, at least, an area of the media world that has a very strong male bias (Lucas and Sherry, 2004). This assumption has, to some degree, fallen out of time, as it is empirically at odds with the fact that now girls and women play digital games at an almost equal rate to boys and men (Vermeulen and Van Looy, 2016; Paaßen et al., 2017; see Melzer, 2018 for an overview).

This does not mean that gender differences in digital games cannot be found. Empirically, women and men differ in what and how they play (e.g., Williams et al., 2009; Greenberg et al., 2010; Poels et al., 2012; Rehbein et al., 2016; Lange and Schwab, 2018; Melzer, 2018). For instance, men play achievement-oriented, competitive and aggressive games more than women do (e.g., Lucas and Sherry, 2004; Hartmann and Klimmt, 2006; Williams et al., 2009; Homer et al., 2012; Hartmann et al., 2015; Puerta-Cortés et al., 2017). Thus, gender is an important factor in the choice of a digital game of a particular genre (Tondello et al., 2018).

Previous research has investigated gender differences in game genre preferences to some extent. However, this existing research has either relied on a small number of digital game genres (e.g., four, namely action/adventure, sports and racing, strategy, and role-playing, as in the research by Thorne et al., 2014), thus over-simplifying the entire phenomenon. Or it has primarily investigated the preferences of North American participants (e.g., Lucas and Sherry, 2004), and, thus, not covered digital games as a worldwide phenomenon (but see Rehbein et al., 2016). However, as a matter of fact, Europe has also a very large digital games market with Germany having the largest among all European countries and the fifth largest worldwide (Newzoo).

**First objective:** The first objective of the current research is, therefore, to examine gender differences in the preferences for (1) a large number of digital game genres and (2) in a European (German) sample. Furthermore, this will be the foundation for our other research objectives.

### Gender Stereotypes on Digital Game Genre Preferences

The previously mentioned belief that primarily boys and men play digital games can be considered a gender stereotype (Vermeulen and Van Looy, 2016). Gender stereotypes in general are assumptions about typical female and male preferences and attitudes combined with the tendency to believe that all or most women and men are a certain way (Jussim et al., 1995; Mealey, 2000; Hentschel et al., 2019).

Although the stereotype that digital games are mostly a male domain is not entirely correct (anymore), digital games still are not stereotype-free. For instance, in digital games, women and men are portrayed in a stereotypical way—not only content-wise (see Lynch et al., 2016) but also, as mentioned above, with respect to whether rather women or men are supposed to play different games (e.g., Hartmann and Klimmt, 2006; Paaßen et al., 2017; Melzer, 2018).

The fact that some genres are considered female while others appear male poses a potential threat to players. If, for instance, a woman prefers shooters, but the stereotypes categorize such games as “male,” the woman might feel obliged to refrain from playing a respective game despite actually liking it. Thus, game genre-related stereotypes might be harmful to players in that they may prevent people from following their actual individual preferences. Given the threat that gaming-related stereotypes might pose to women in particular (see, for instance, Kaye and Pennington, 2016; Vermeulen et al., 2016), it is important to understand the gender stereotypes that exist with respect to digital games.

**Second objective:** The second objective of the current research is, therefore, to investigate gender stereotypes on digital game genre preferences.

### The Accuracy of Gender Stereotypes on Digital Game Genre Preferences

The sheer fact that stereotypes can be harmful does not necessarily mean that they are entirely wrong. Empirically, stereotypes do reflect, at least to some degree, real differences between groups (Hilton and von Hippel, 1996; Jussim et al., 2009; Brown, 2010). The question of whether a stereotype is accurate (in that it reflects an empirically evident mean difference between groups) is, thus, an empirical rather than an ideological one (Jussim et al., 2009).

Previous empirical research has focused on whether, and, if so, to what degree gender stereotypes actually do reflect reality (Swim, 1994; Jussim et al., 2009). This research on the accuracy of gender stereotypes has found, for instance, that respective gender stereotypes about cognitive abilities are basically correct, but underestimate the actual gender differences in some domains (Halpern et al., 2011) while overestimating them in others (e.g., verbal abilities; Swim, 1994).

Despite gender stereotype accuracy research in general (e.g., Jussim et al., 2009), almost no previous studies on gender stereotypes have addressed media preferences. A recent analysis of movie genre preferences by Wühr et al. (2017) constitutes an exception. They assessed (1) gender stereotypes on movie genre preferences of women and men, (2) actual gender differences in movie genre preferences, and (3) compared these findings with each other. They found that gender stereotypes in movie genre preferences were mostly accurate in direction, but inaccurate in size for the majority of the genres in that they overestimated the actual gender differences. The authors were, thus, able to provide an insightful and differentiated, though also ambivalent, picture of the validity of gender stereotypes with respect to media preferences.

The question now arises, whether the findings of Wühr et al. (2017) are only valid for a relatively classic medium (i.e., movies) or whether there is a relatively robust pattern across different media, including a popular medium like digital games, according to which gender stereotypes are basically accurate in direction but not necessarily in size. Those two aspects constituted the third objective of the current study with a focus on digital games.

**Third objective:** The third objective of the current research is, therefore, to investigate the accuracy of the game genre-related gender stereotypes by comparing them to the actual gender differences in digital game genre preferences.

Study 1 was designed to assess the effects of gender (first objective) on preferences for digital game genres. In Study 2, we assessed beliefs on how typically female or male these video game genres are (i.e., gender stereotypes; second objective). Finally, by comparing the results from Study 1 to those of Study 2, we wanted to assess the direction and accuracy of gender stereotypes of digital game genres (third objective).

This analysis gives us a complete picture about how large gender differences in digital game genre preferences are and how large they are thought to be. Our research was exploratory in that we did not propose hypotheses but only research objectives. Based on previous research (e.g., Wühr et al., 2017), it would have been possible to make relatively clear predictions a-priori. However, we wanted to approach the topic more open-mindedly instead of being too fixed in what to expect. The exploratory nature of our research was also the reason (1) why we did not conduct an a-priori power analysis to determine the sizes of our samples and (2) why we did not correct for an alpha error inflation. As there is not much media-related gender stereotype research—to our best knowledge, the study by Wühr et al. (2017) is the only one—we decided not to adjust significance values (e.g., by means of a Bonferroni correction) to make sure not to miss important results (cf. Rothman, 1990; Perneger, 1998).

## Study 1: Effects of Gender on Digital Game Genre Preferences

First, we checked for gender differences in digital game genre preferences (first objective).

### Methods

In Study 1, a total number of 484 participants (203 women) between the ages of 14–64 years (*M* = 23.88, *SD* = 5.35) completed an online survey in German. Participants were recruited by the first author by postings on social network sites (e.g., Facebook). Involvement in the study was voluntary and not compensated. All participants gave informed consent. The research reported in our manuscript meets the ethical guidelines of the German Society of Psychology (Deutsche Gesellschaft für Psychologie, DGPs) and is consistent with the principles of research ethics as published by the American Psychological Association (APA). Data collection was completely anonymous. That is, except for age and gender, we did not record personal information from the participants. The research reported here had not been approved by a local ethics committee because the ethical guidelines of the German Society of Psychology do not require ethical approval of basic psychological studies involving simple behavioral data.

The questionnaire included 17 genre items, one item per genre. Participants were presented the following statement for each genre: “I like to play [game genre] ([specification of one genre]), for instance [*concrete example(s) for some genres*].” One statement, for instance, was as follows: “I like to play adventure games (action, action-adventure), for instance, *Grand Theft Auto, The Legend of Zelda, Tomb Raider, Devil May Cry, Resident Evil*.” The participants were asked to indicate how much they agree with each of the statements on a five-point rating scale ranging from 1 = disagree to 5 = agree.

The game genres were [specification of genre/*example(s) for genre* provided to the participants in parentheses]: Adventure games (action, action-adventure/*Grand Theft Auto, The Legend of Zelda, Tomb Raider, Devil May Cry, Resident Evil*); Beat ‘em ups (*Tekken, Street Fighter, Virtua Fighter*); Casual games (*Frogger, Tetris*); Educational games; Erotic games (*Leisure Suit Larry*); First- and Third-Person shooters (*Call of Duty, Battlefield, Metal Gear Solid*); Music and dance games (*Guitar Hero, Rock Band, Dance Dance Revolution, Just Dance*); Open-world games (*Minecraft*); Platformers (*Super Mario, Sonic*); Puzzle games (*Lemmings*); Quiz games; Role-playing games (*Final Fantasy, Pokémon, Dragon Quest*); Shoot ‘em ups (*Zaxxon, Duke Nukem, House of the Dead, Virtua Cop, Time Crisis*); Simulation games (*The Sims, SimCity*); Sports games (*Madden NFL, Wii Sports, Tiger Woods PGA Tour, FIFA, Sega Rally, Need For Speed*); Strategy games (*Age of Empires, Warcraft, Civilization*), and Western games (*Red Dead Redemption*). The choice of those 17 genres (including specifications and examples) was inspired by the German and English Wikipedia sites on video game genres. To be precise, we carefully read the respective sites and checked which genres were listed there. Then we chose some of the games that were listed for each genre as examples.

The questionnaire also included items on demographics (age and gender) as well as an item on whether the participant considered her or himself as a gamer (yes or no). Also, an item was included asking for how many hours a week the participants played digital games. The last two items were included in order to investigate whether gender differences in gaming habits exist.

### Results

**Figure 1:**
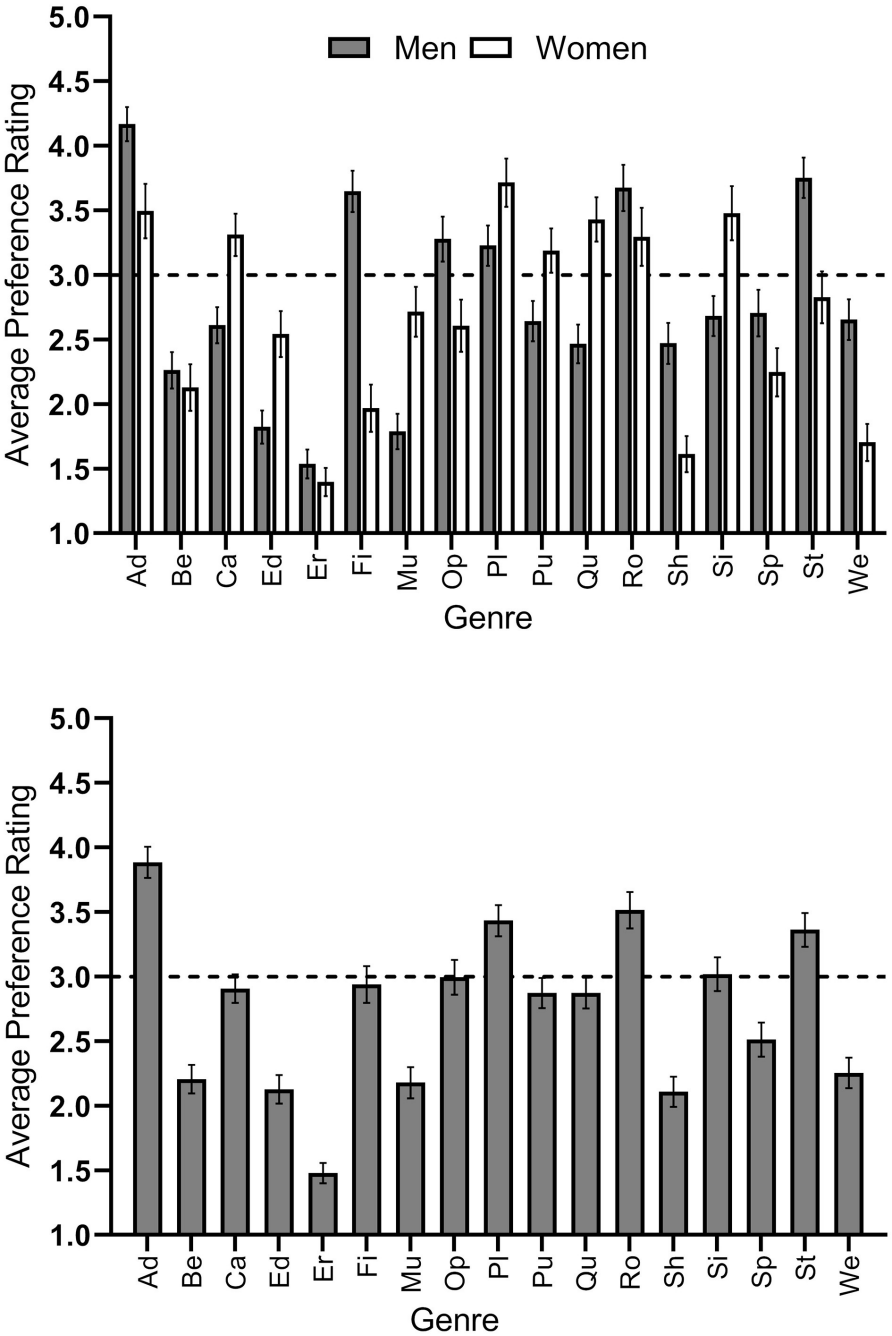
Average ratings from participants who indicated their preferences for 17 digital game genres on a 5-point rating scale ranging from 1 = disagree to 5 = agree by gender of the participant **(Top)** and total **(Bottom)**. The grid line at Y = 3 indicates the mid-point of the scale. Bars above the line indicate a preferred genre. Error bars show the 95% confidence intervals of the means. Abbreviations on the x-axis stand for: Ad, adventure; Be, beat ‘em up; Ca, casual; Ed, educational; Er, erotic; Fi, first- and third-person shooter; Mu, music and dance; Op, open-world; Pl, platformer; Pu, puzzle; Qu, quiz; Ro, role-playing; Sh, shoot ‘em up; Si, simulation; Sp, sports; St, strategy; We, western.

Independent of gender and other variables, adventure was the genre that was preferred the most (*M* = 3.82, *SD* = 1.27). Five genres received average ratings of three or above and were, therefore, preferred rather than unpreferred. See Figure 1 (bottom) for a visual summary. Next, we checked for gender differences in the self-labeling gamer distinction. Most male participants (85.4%) considered themselves gamers, while only a minority of female participants (45.8%) did [${\text{X}}_{\left(1\right)}^{2}$ = 86.09, *p* < 0.001]. Accordingly, male participants played for significantly more hours a week (*M* = 11.89, *SD* = 12.88) than female participants [*M* = 4.91, *SD* = 8.93; *t*_(459.982)_ = 6.883, *p* < 0.001, *d* = 0.64].

As to the genre preferences (first objective: Gender differences in digital game genre preferences), seven genres received average ratings of three or above by women and were thus considered to be preferred by them. The favorite female genre was platformer (*M* = 3.72, *SD* = 1.30). Six genres received average ratings of three or above by men and were thus considered preferred by them. The favorite male genre was adventure (*M* = 4.17, *SD* = 1.07). See Figure 1 (top) for a visual summary.

Genres more preferred by women than by men were (effect size Cohen's *d* in parentheses): music and dance (−0.76), quiz (−0.66), educational (−0.63), casual (−0.62), simulation (−0.59), puzzle (−0.45), and platformer (−0.38). Genres more preferred by men than by women were: first- and third-person shooters (1.31), western (0.84), shoot ‘em ups (0.76), strategy (0.69), adventure (0.54), open-world (0.48), sports (0.33), and role-playing (0.25).

According to the effect size categorization by Cohen (1988), gender differences were, thus, large for two genres, moderate for eight genres, small for five genres, and non-existent for two genres (beat ‘em up, *d* = 0.13; erotic, *d* = 0.17). The largest gender difference was found for first- and third-person shooter. Men (*M* = 3.65, *SD* = 1.30) expressed a relatively large preference for this genre, whereas women did not seem to like that genre very much (*M* = 1.97, *SD* = 1.26, *d* = 1.31; see above). The smallest gender differences (beat ‘em up and erotic) were less than *d* = 0.20 (see above) and thus negligible (Cohen, 1988). Hence, we considered those two genres to be gender-neutral. Table 1 provides the respective values.

**Table 1:** Average ratings (*M, SD*) from participants who indicated their preferences for 17 digital game genres on a five-point rating scale ranging from 1 = disagree to 5 = agree by gender of the participant (including *t, p*, and *d* values for gender differences and preference categorization) and total.

**Genre**	**Men**		**Women**		**Actual difference**	**Actual preference**	**Total**	
	***M***	***SD***	***M***	***SD***	***t, p, d***		***M***	***SD***
Ad	4.13	1.08	3.50	1.46	5.17, <0.001, 0.50	Male	3.86	1.29
Be	2.27	1.15	2.11	1.25	1.40,0.162, 0.13	Neutral	2.20	1.20
Ca	2.59	1.16	3.25	1.14	−6.13, <0.001, −0.57	Female	2.87	1.20
Ed	1.83	1.03	2.54	1.23	−6.58, <0.001, −0.62	Female	2.13	1.17
Er	1.54	0.91	1.37	0.73	2.26,0.025, 0.21	Male	1.47	0.84
Fi	3.61	1.29	1.99	1.28	13.64, <0.001, 1.27	Male	2.93	1.51
Mu	1.79	1.11	2.69	1.33	−7.76, <0.001, −0.74	Female	2.17	1.29
Op	3.25	1.41	2.60	1.39	4.92, <0.001, 0.46	Male	2.98	1.43
Pl	3.21	1.28	3.70	1.30	−4.12, <0.001, −0.38	Female	3.42	1.31
Pu	2.62	1.25	3.16	1.19	−4.81, <0.001, −0.45	Female	2.85	1.25
Qu	2.46	1.22	3.36	1.23	−7.95, <0.001, −0.74	Female	2.84	1.30
Ro	3.67	1.42	3.27	1.56	2.85,0.005, 0.27	Male	3.50	1.49
Sh	2.50	1.30	1.62	0.98	8.43, <0.001, 0.77	Male	2.13	1.25
Si	2.68	1.25	3.48	1.45	−6.27, <0.001, −0.59	Female	3.01	1.39
Sp	2.72	1.47	2.24	1.28	3.76, <0.001, 0.35	Male	2.52	1.41
St	3.76	1.26	2.84	1.41	7.37, <0.001, 0.69	Male	3.38	1.40
We	2.64	1.28	1.69	0.98	9.20, <0.001, 0.85	Male	2.24	1.25

*Negative t and d values indicate a stronger female preference, whereas positive values indicate a stronger male preference. As to the preference categorization, d values of at least 0.20 were used as threshold values. Short-hands for column “Genre:” Ad, adventure; Be, beat ‘em up; Ca, casual; Ed, educational; Er, erotic; Fi, first- and third-person shooter; Mu, music and dance; Op, open-world; Pl, platformer; Pu, puzzle; Qu, quiz; Ro, role-playing; Sh, shoot ‘em up; Si, simulation; Sp, sports; St, strategy; We, western*.

The mean effect size for the gender differences in preferences for digital game genre preferences across all genres was *d* = 0.56. Figure 1 (top) gives a visualization of the respective findings.

Additionally, we conducted a discriminant analysis in order to assess the accuracy in identifying participants' gender based only on their preferred game genres. The resulting function discriminated very well between gender of participants [Wilks's λ = 0.52, ${\text{X}}_{\left(17\right)}^{2}$ = 279.17, *p* < 0.001]. Women were identified as women with an accuracy of 83.9% and men as men with an even higher accuracy of 85.5% only from the information of genre preferences. Furthermore, it became evident which genres were actually discriminant. Beat ‘em up was not significant in this respect (*p* = 0.243), Erotic was only marginally significant (*p* = 0.087). All other genres were highly significant (*p*s < 0.001).

Finally, we conducted an exploratory multivariate ANOVA with the genre ratings as DVs and gender as well as “gamer” (yes / no) as IVs to test for a possible confound between gender and self-labeling as gamer. Most importantly, this analysis revealed no significant gender x gamer interaction (*Pillai's trace V* = 0.056, *F* = 1.50, *p* = 0.09) (more details about this analysis can be obtained from the first author upon request).

### Discussion

Research has shown that, for instance, men play achievement-oriented, competitive, and aggressive games more than women do (e.g., Williams et al., 2009). These findings are similar to those for other media preferences (Goldstein, 1999), such as movies (e.g., Krcmar and Kean, 2005; see Wühr et al., 2017 for an overview). Our results on digital games are in accordance with these former findings. Some of the numerous gender differences in game genre preferences that we found were remarkably large, which is also basically in line with previous findings (Lucas and Sherry, 2004). Given the fact that research has consistently shown that men, contrary to women, like violent and competitive games (Lucas and Sherry, 2004; Hartmann and Klimmt, 2006; Williams et al., 2009; Hartmann et al., 2015; Puerta-Cortés et al., 2017) and that this gender difference is evident as early as at the age of 9 years (von Salisch et al., 2011), this gaming-related gender difference can be considered a robust phenomenon. However, not all genres preferred by men in our study are exclusively violent (e.g., strategy, role-playing).

## Study 2: Stereotypes on Gender Differences in Digital Game Genre Preferences

Next, we examined the stereotypes about digital game genre preferences of women and men (second objective) and assessed the accuracy of these stereotypes (third objective).

### Methods

In Study 2, a total number of 228 participants between the ages of 17–60 years (*M* = 24.12, *SD* = 6.35) filled out an online survey in German. Among them were 125 women. One person responded as neither female nor male, and one person did not want to answer. As the research investigated gender differences, those two respondents were excluded prior to conducting the statistical analyses (*N* = 226). The participants were recruited by the first author by postings on social network sites (e.g., Facebook). Participation was voluntary and not compensated. All participants gave informed consent. Similar to Study 1 (see above), we have complied with APA ethical standards in the treatment of our sample.

The questionnaire included items on the same 17 genres described above. The participants were asked to rate how much women/men like certain video game genres. The following questions were asked for each genre: “What do you think? Are [game genre] ([specification of one genre]), for instance [concrete example(s) for some genres] preferred more by women or by men?” One item, for instance, was as follows: “What do you think? Are adventure games (action, action-adventure), for instance *Grand Theft Auto, The Legend of Zelda, Tomb Raider, Devil May Cry, Resident Evil*, preferred more by women or by men?” (cf. above). Participants had to indicate their answer on a five-point rating scale from 1 = exclusively preferred by women to 5 = exclusively preferred by men. The mid-point of the scale (3) was marked as “equally preferred by women and men.”

Again, the questionnaire included items, among others, on demographics (age and gender) as well as the aforementioned item on how many hours per week the participants play digital games.

### Results

First, we will present the results for assumed gender differences (gender stereotypes; second objective) followed by a comparison of those gender stereotypes to the actual gender differences from Study 1 in order to check for stereotype accuracy (third objective).

#### Second Objective: Gender Stereotypes in Digital Game Genre Preferences

**Figure 2:**
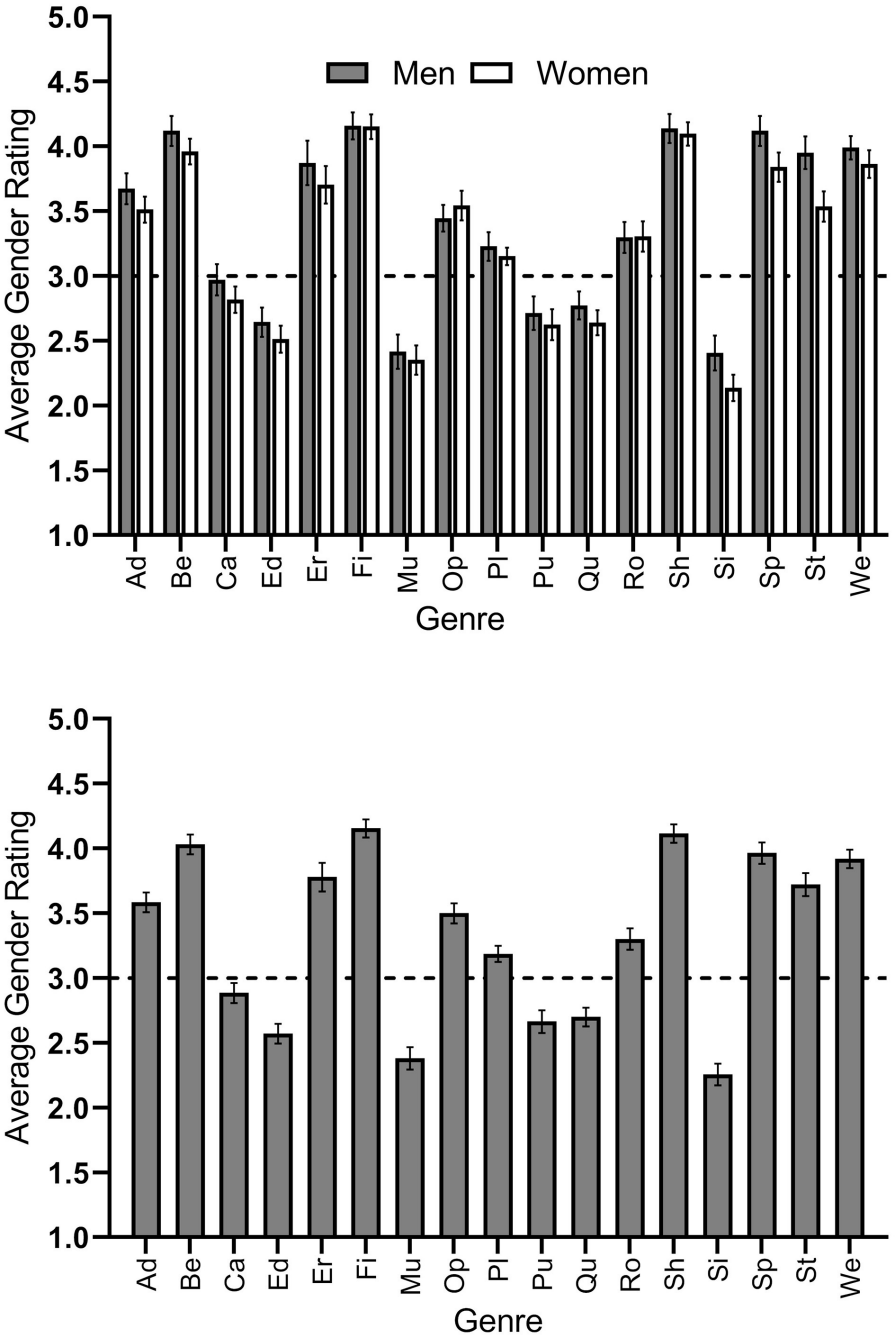
Average ratings from participants who indicated the degree to which 17 digital game genres were presumably preferred by women or men on a 5-point rating scale ranging from 1 = exclusively preferred by women to 5 = exclusively preferred by men by gender of the participant **(Top)** and total **(Bottom)**. The grid line at Y = 3 indicates the mid-point of the scale. Bars below the line indicate a stronger presumed female preference for a genre, whereas bars above the line indicate a stronger presumed male preference. Error bars show the 95% confidence intervals of the means. Abbreviations on the x-axis mean: Ad, adventure; Be, beat ‘em up; Ca, casual; Ed, educational; Er, erotic; Fi, first- and third-person shooter; Mu, music and dance; Op, open-world; Pl, platformer; Pu, puzzle; Qu, quiz; Ro, role-playing; Sh, shoot ‘em up; Si, simulation; Sp, sports; St, strategy; We, western.

First, we computed the means of the ratings of our female and male participants (aggregated data). Thus, with the 17 genres as our cases, we had a female and a male mean value for each genre. With respect to this data, our female and male participants highly agreed on what genres women and men presumably prefer, as their ratings highly correlated with each other (*r* = 0.98, *p* < 0.001; see Figure 2 top). Hence, for estimating stereotype accuracy, we used the total values each genre received (aggregated data) for presumed gender differences (see Figure 2 bottom).

When investigating the values on a descriptive level, six genres received ratings below three and were thus considered to be preferred by women. The genre that was presumed to be preferred the most by women was simulation (*M* = 2.26, *SD* = 0.64). Eleven genres received ratings of three or above and were thus considered to be preferred by men. The genre presumed to be most preferred by men was first- and third-person shooter (*M* = 4.15, *SD* = 0.53). See Figure 2 (bottom) and Table 2 for an overview.

**Table 2:** Average ratings from participants who indicated the degree to which 17 digital game genres are presumably preferred by women or men on a 5-point rating scale ranging from 1 (exclusively preferred by women) to 5 (exclusively preferred by men), by gender of the participant and total (including *t, p*, and *d* values for gender differences and preference categorization).

**Genre**	**Men**		**Women**		**Total**		**Presumed difference**	**Presumed preference**
	***M***	***SD***	***M***	***SD***	***M***	***SD***	***t, p, d***	
Ad	3.67	0.60	3.51	0.56	3.58	0.59	15.019, <0.001, 1.00	Male
Be	4.12	0.59	3.96	0.56	4.03	0.58	26.884, <0.001, 1.79	Male
Ca	2.97	0.61	2.82	0.57	2.88	0.59	−2.919,0.004, −0.19	Neutral
Ed	2.64	0.58	2.51	0.59	2.57	0.59	−11.004, <0.001, −0.73	Female
Er	3.87	0.87	3.70	0.81	3.78	0.84	13.930, <0.001, 0.93	Male
Fi	4.16	0.52	4.15	0.54	4.15	0.53	32.660, <0.001, 2.17	Male
Mu	2.42	0.67	2.35	0.64	2.38	0.65	−14.311, <0.001, −0.95	Female
Op	3.45	0.52	3.54	0.64	3.50	0.59	12.726, <0.001, 0.85	Male
Pl	3.23	0.56	3.15	0.38	3.19	0.47	5.915, <0.001, 0.39	Male
Pu	2.71	0.65	2.62	0.67	2.66	0.66	−7.643, <0.001, −0.51	Female
Qu	2.77	0.55	2.64	0.55	2.70	0.55	−8.256, <0.001, −0.55	Female
Ro	3.30	0.61	3.30	0.66	3.30	0.64	7.092, <0.001, 0.47	Male
Sh	4.14	0.57	4.10	0.52	4.12	0.54	31.185, <0.001, 2.07	Male
Si	2.41	0.68	2.14	0.57	2.26	0.64	−17.561, <0.001, −1.17	Female
Sp	4.12	0.59	3.84	0.64	3.96	0.63	22.964, <0.001, 1.53	Male
St	3.95	0.64	3.54	0.65	3.72	0.68	15.991, <0.001, 1.06	Male
We	3.99	0.46	3.86	0.60	3.92	0.54	25.437, <0.001, 1.69	Male

*Negative t and d values indicate a stronger presumed female, whereas positive values indicate a stronger presumed male preference. As to the preference categorization, d values of at least 0.20 were used as threshold values. Short-hands for column “Genre:” Ad, adventure; Be, beat ‘em up; Ca, casual; Ed, educational; Er, erotic; Fi, first- and third-person shooter; Mu, music and dance; Op, open-world; Pl, platformer; Pu, puzzle; Qu, quiz; Ro, role-playing; Sh, shoot ‘em up; Si, simulation; Sp, sports; St, strategy; We, western*.

As we had effect sizes for gender differences in actual genre preferences from Study 1 (see Table 1), it was essential to check the effect sizes for presumed gender differences in genre preferences (see Table 2). Genres presumed to be more preferred by women were (effect size Cohen's *d* in parentheses): simulation (−1.17), music and dance (−0.95), educational (−0.73), quiz (−0.55), and puzzle (−0.51). Genres presumed to be more preferred by men were: first- and third-person shooter (2.17), shoot ‘em up (2.07), beat ‘em up (1.79), western (1.69), sports (1.53), strategy (1.06), adventure (1.00), erotic (0.93), open-world (0.85), role-playing (0.47), and platformer (0.39). See Table 2 for an overview.

According to the effect size categorization by Cohen (1988), presumed gender differences were large for 11 genres, moderate for four genres, small for one genre, and non-existent for one genre (casual; *d* = −0.19; cf. above; see also Table 2). The largest presumed gender difference was found for first- and third person shooter. Our participants gave an average rating of *M* = 4.16 (*SD* = 0.52) on the 5-point rating scale with 5 having been “exclusively preferred by men” (*d* = 2.17). The average effect size for presumed gender differences was *d* = 1.06. Figure 2 provides a visual summary of the findings for presumed gender differences. Table 2 provides the mean values each genre received on presumed gender differences with those mean values being tested against the mid-point of the scale (i.e., 3) using one-sample *t*-tests.

#### Third Objective: The Accuracy of Gender Stereotypes on Digital Game Genre Preferences

In order to investigate the accuracy of gender stereotypes on game genre preferences, we first compared the directions of the means of the actual (Study 1) and presumed (Study 2) gender differences.

The means went in opposite directions for one genre only (platformer). While women (*M* = 3.70) expressed a higher preference for this genre than did men (*M* = 3.21; see Table 1), our participants of Study 2 presumed that this genre was “male” (*M* = 3.19; see Table 2). Therefore, in this respect, gender stereotypes were correct for 16 out of 17 genres.

However, using only the directions of the means might not be an adequate measure for stereotype accuracy, as this does not account for the size of an actual or a presumed gender difference. We hence categorized a genre as “female,” if it went at least with a small effect into the female direction (*d* ≤ −0.20); we used “male,” if it went at least with a small effect into the male direction (*d* ≥ 0.20; cf. Cohen, 1988); otherwise, we considered it a neutral genre. Thus, we compared actual gender differences to the ones in presumed preferences using categorial variables (male, female, neutral; see Tables 1, 2). According to this procedure, 13 out of 17 genre stereotypes were still correct. For only the following four genres, the stereotypes were not correct: beat ‘em up (no actual gender difference, but presumed to be preferred by men), casual (preferred by women, but presumed to be a neutral genre), erotic (no actual gender difference, but presumed to be preferred by men) and platformer (preferred by women, but presumed to be preferred by men). See Tables 1, 2 for an overview and for a comparison between actual and presumed gender differences.

Such categorization does not account for over- or underestimations of gender differences though. We hence calculated the differences between the effect sizes for the actual and the presumed gender differences. Table 3 provides an overview of the results.

**Table 3:** Effect sizes (*d*) for actual gender differences in genre preferences (1), presumed gender differences in genre preferences (2), and for the accuracy of the gender stereotypes (3).

**Genre**	**(1) Actual gender difference**	**(2) Presumed gender difference**	**(3) Accuracy of gender stereotype (1 vs. 2)**	
Ad	0.50	1.00	|0.50|	(++)
Be	0.13	1.79	|1.66|	(+++)
Ca	−0.57	−0.19	|0.38|	(–)
Ed	−0.62	−0.73	|0.11|	
Er	0.21	0.93	|0.72|	(++)
Fi	1.27	2.17	|0.91|	(+++)
Mu	−0.74	−0.95	|0.21|	(+)
Op	0.46	0.85	|0.39|	(+)
Pl	−0.38	0.39	|0.78|	(x)
Pu	−0.45	−0.51	|0.06|	
Qu	−0.74	−0.55	|0.19|	
Ro	0.27	0.47	|0.20|	(+)
Sh	0.77	2.07	|1.30|	(+++)
Si	−0.59	−1.17	|0.58|	(++)
Sp	0.35	1.53	|1.18|	(+++)
St	0.69	1.06	|0.37|	(+)
We	0.85	1.69	|0.85|	(+++)

*Negative d values indicate a higher actual or presumed female preference, whereas positive values indicate a stronger actual or presumed male preference. Column (3): In parentheses, categorizations of over- / underestimations are given: +, small; ++, moderate; +++, large overestimation; –, small underestimation (cf. Cohen, 1988); x, no correct estimation. Short-hands for column “Genre:” Ad, adventure; Be, beat ‘em up; Ca, casual; Ed, educational; Er, erotic; Fi, first- and third-person shooter; Mu, music and dance; Op, open-world; Pl, platformer; Pu, puzzle; Qu, quiz; Ro, role-playing; Sh, shoot ‘em up; Si, simulation; Sp, sports; St, strategy; We, western*.

**Figure 3:**
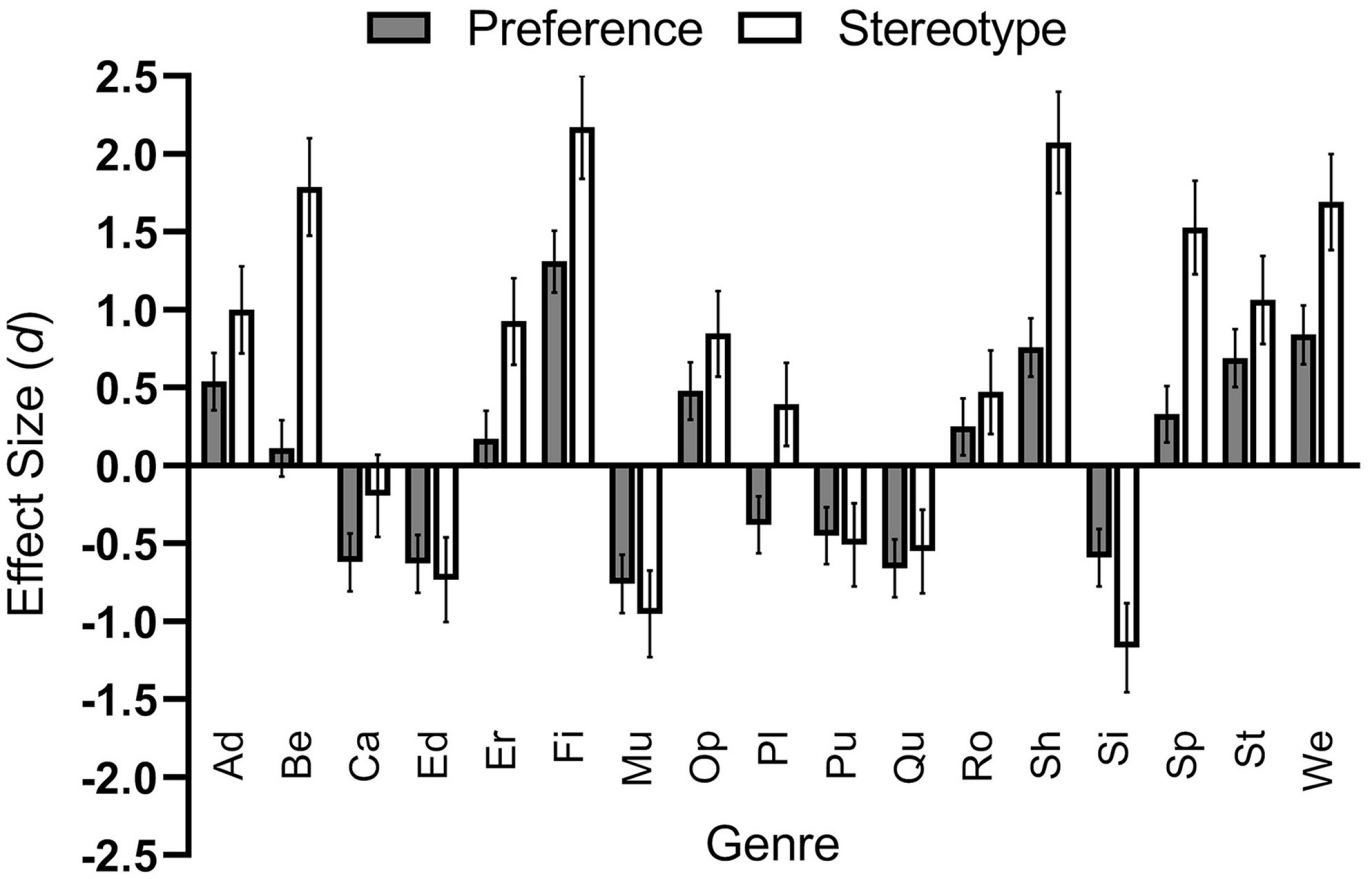
Comparison between actual and presumed gender differences with respect to digital game genre preferences. Gray bars show the effect sizes (Cohen's *d*) of the actual gender differences with respect to digital game genre preferences. Negative values indicate a stronger female preference for a genre, whereas positive values indicate a stronger male preference. White bars show the effect sizes (Cohen's *d*) of the presumed gender differences with respect to digital game genre preferences. Negative values indicate a stronger presumed female preference for a genre, whereas positive values indicate a stronger presumed male preference. Error bars show the 95% confidence intervals of the *d* values. Abbreviations on the x-axis mean: Ad, adventure; Be, beat ‘em up; Ca, casual; Ed, educational; Er, erotic; Fi, first- and third-person shooter; Mu, music and dance; Op, open-world; Pl, platformer; Pu, puzzle; Qu, quiz; Ro, role-playing; Sh, shoot ‘em up; Si, simulation; Sp, sports; St, strategy; We, western.

As can be seen in Table 3, gender differences in game genre preferences, although mostly correct in direction, were strongly overestimated. For instance, for five genres, the actual gender difference was overestimated with a large effect size. Only for one genre (casual) was the actual gender difference (with a small effect) underestimated by the stereotype (*d* = 0.43). For four genres (educational, music and dance, puzzle, quiz), the stereotypes matched the actual gender differences relatively well (*d*s < 0.20). As stated above, for platformer, the actual gender difference and the stereotype went in opposite directions. For all genres that were correct in direction but over- or underestimated the actual gender differences with at least a small effect size (*d*s ≥ 0.20; *n* = 12), actual gender differences were overestimated by the stereotype with an average *d* of 0.76. Taking all genres into consideration (except for platformer, for which the ratings went in opposite directions; thus, *n* = 16), the overestimation was still moderate with *d* = 0.60. Figure 3 provides a visual summary of these findings.

As can be seen, gender differences in actual preferences are mostly moderate in size, while the presumed gender differences in genre preferences are mostly quite large.

### Discussion

It became evident that, very broadly speaking, the stereotypes are basically accurate; however, when taking a more profound and closer look at it, they overestimate the actual gender differences. A similar finding was evident for movie genre preferences (Wühr et al., 2017). Some previous studies have also found that gender stereotypes overestimate actual gender differences (e.g., Martin, 1987; Allen, 1995), although other studies demonstrate that gender stereotypes might also underestimate real gender differences (e.g., McCauley and Thangavelu, 1991; Halpern et al., 2011).

## General Discussion

In Study 1, we found numerous large actual gender differences in game genre preferences. As for an explanation of the obtained gender differences, differences in gender identity constitute one possible explanation (Poels et al., 2012); social constructionist approaches are another (Vermeulen and Van Looy, 2016; see also Lucas and Sherry, 2004). Also, social roles theory (Eagly, 1987; see also Steinmetz et al., 2014; Eagly and Sczesny, 2019) offers a framework for investigating gender. The effects of prenatal testosterone constitute yet another explanation (Mazur et al., 1997; Huh, 2011; see also Constantinescu and Hines, 2012). Those explanations, however, only consider proximate mechanisms (cultural- / environmental-proximate in the first cases, biological-proximate in the latter case).

Another explanation takes an evolutionary perspective (e.g., Lueptow et al., 1995; Zhu and Chang, 2019; see also Mealey, 2000). It is not necessarily at odds with, and even in accordance with the other explanations, but adds the ultimate ‘why’ question (cf. Wühr et al., 2017). This so-called ultimate perspective asks for which functional reasons men, for instance, evolved to show a certain propensity to action, violence, and the like on average, and why women, on average, tend to be more apt to engage in social activities. This perspective, which complements rather than contradicts the accounts mentioned before, asks: Why (in terms of functionality) do men anticipate more enjoyment from violent games? And why do men score higher (cross-culturally) on several dimensions of aggression?

There is in fact a growing body of theoretical and empirical research that adopts this ultimate perspective and tries to reconcile it with the aforementioned proximate explanations on digital games (e.g., Mendenhall et al., 2010a,b; Bertozzi, 2014; Kasumovic et al., 2015; Devilly et al., 2017; Breuer, 2018a; Brill et al., 2018; Lange and Schwab, 2018; Lange et al., 2018; Liebold et al., 2018; Lynch, 2018; Melzer, 2018), other media or the media in general (e.g., Ratnasingam and Ellis, 2011; Hennighausen and Schwab, 2015; Brill and Schwab, 2020), as well as on human culture in general (e.g., Lange et al., 2013). From this perspective, the underlying reasons for gender differences in media use, selection (based on preferences), and effects can, to some degree, be attributed to mate choice. Due to gender differences in so-called obligatory parental investment (Trivers, 1972), mate choice in humans is female mate choice, which causes men to have a particularly strong intra-sexual competition including violence (Wilson and Daly, 1985). Research on the motivation for playing violent digital games has indeed confirmed this assumption (Kasumovic et al., 2015).

To summarize, it seems likely that an on average higher male competitive preference is the result of evolutionary selection pressures (ultimate) and its corresponding biological correlates (proximate-biological). This propensity can, possibly to a large degree, be moderated by environmental factors (proximate-sociocultural) (Croson and Gneezy, 2009). This competitive preference is at work when it comes to the selection of a digital game of a particular genre. Of course, this does not mean that all men prefer competitive games; rather, there are varying degrees of femininity and masculinity within each of the genders (Poels et al., 2012). Still, the simple dichotomy of female-male turned out to be a very strong predictor of game genre preferences. In line with this finding, other research has demonstrated that humans are able to guess people's dichotomous gender very accurately only based on, for instance, small portions of linguistic material that those people produced (e.g., Lange et al., 2016).

Furthermore, we also found numerous—and even larger—presumed gender differences, that is gender stereotypes, for digital game genre preferences (Study 2). Those gender stereotypes were not entirely wrong. As a matter of fact, they went mostly in the correct direction. However, they substantially overestimated the actual gender differences.

As for the reason behind this overestimation, it seems worthwhile to dive into the actual functionality of stereotypes, namely to cognitively order a vast variety of social and other information bombarding our senses (Ashmore and Del Boca, 1981; Hamilton and Sherman, 1994). This ordering process is conducted by building categories. It might be that the categories in our minds are much more distinct than the respective differences in real life. Thus, women and men are assigned to two different categories much more strongly than they are actually apart from each other.

This might be problematic because it could lead to the exclusion of girls and women from certain gaming communities or maybe even add to the proliferation of misogyny and sexism in general and in these communities in particular (for overviews on and a discussion of gaming-related misogyny, sexism, and other forms of stigmatization, see Kowert et al., 2017; Vermeulen et al., 2017; Breuer, 2018b; Tompkins and Lynch, 2018; Wasserman and Rittenour, 2019). The conclusion could be to try make gamers aware of the actual size of gender differences (i.e., that women and men are less different than what people think or what is portrayed in the games) in order to avoid harmful effects. Also, video game designers should try to portray women and men in games in a less stereotypical way.

### Limitations

In our research, we were not investigating actual and presumed gender differences in preferences for, for instance, competitive games. Instead, following previous research on gender stereotype accuracy of media preferences (Wühr et al., 2017), we were focusing on genres, as other research has previously done (e.g., Thorne et al., 2014). The 17 genres we had were taken from the sites of the well-known online dictionary *Wikipedia* and appeared to be face-valid. We were confident that opting for 17 genres would work, as similar former research (on movie genre preferences; Wühr et al., 2017) had the same number. A far smaller number of genres (e.g., four, in the research by Thorne et al., 2014), was no option for us, as this would have resulted in a too simplistic view on the variety of different types of games. Still, referring to *Wikipedia* for our genre classification can be considered a limitation. *Wikipedia* articles are probably quite often written by laypeople and might, thus, suffer from a lack of rigor. Future studies on genre might, hence, use different approaches on how to obtain a proper genre classification (e.g., by using a panel of experts).

Apart from that, relying on any genre classification is problematic, at least to some degree (Clarke et al., 2017; Faisal and Peltoniemi, 2018). For instance, simulation can mean life simulation (presumably preferred by women) or combat simulation (presumably preferred by men) (cf. Rehbein et al., 2016). Therefore, the content of a certain genre term can be fuzzy. We were, however, trying to avoid problems of this kind by providing our participants with concrete examples of games for the different genres. For simulation, we referred to *The Sims* (a life simulation) and *SimCity* (a construction/management simulation). Also, the western genre seems to be somewhat a niche genre compared to, for instance, open-world games or platformers. Furthermore, not all participants in our study might have been equally familiar with game genres. However, as stated above, we were as concrete as possible in terms of how the genres were presented, as we not only used the names of the game genres, but also provided specifications of the genres and named actual games as examples. Still, it might have been that some participants were unsure about how to respond.

Furthermore, we did not explicitly include, for instance, eSports (i.e., playing digital games in competitive tournaments and the like), as we wanted to focus on classical digital gaming. However, future studies on gaming preferences should also consider eSports as one growingly important part of gaming culture (for more information on eSports, see Cunningham et al., 2018; Pizzo et al., 2018; Darvin et al., 2020). However, our research might have some implications for eSports as well. Consider, for example, a female gamer looking to join an eSports team (“clan”) in a male-biased genre (e.g., first- and third person shooter). Gender stereotypes could hinder this female gamer to join this team. On the other hand, team members may also be skeptical concerning the skills of this female gamer. Finally, the audience may also be skeptical if team members violate the gender stereotypes of this particular genre. More research is needed to investigate this directly.

Also, we were focusing on binary gender (female and male), thus excluding any non-binary gender classification. However, in Study 2, apart from a “female” and a “male” option, we offered the option to our participants to choose “neither female nor male” or to refuse to answer. In fact, only one out of 228 participants (0.44%) chose the “neither female nor male” option. In line with this finding, other research has demonstrated that almost 100% of all people are clearly either female or male (cf. Sax, 2002). Furthermore, any non-binary gender approach was beyond our scope to start with. Still, it could we worthwhile to study media preferences of people not identifying themselves clearly as either female or male.

Furthermore, our two study samples were only convenience samples. This might have influenced our results to some degree. However, using convenience samples is quite common in social science research.

Finally, our research was exploratory. Future research of this kind might (1) propose hypotheses instead of research questions or objectives and (2) adjust for alpha inflation.

### Conclusion

In summary, we found numerous gender differences in game genre preferences. These were much larger than many gender differences in other domains (Hyde, 2005). When it comes to media selection, gender could be a particularly strong predictor (see also Wühr et al., 2017).

Furthermore, strong stereotypes on women's and men's game genre preferences exist. Despite the potential harm that those stereotypes can cause, they were not entirely wrong, although they overestimated the actual gender differences by a relatively large margin. This is the first finding of this kind for digital games that is, furthermore, in line with the respective findings for movies (Wühr et al., 2017). Further research needs to focus on the reasons for the strong overestimations of actual gender differences (in games as well as in movies), that is for the somewhat exaggerated gender stereotypes.

## Data Availability Statement

The raw data supporting the conclusions of this article will be made available by the authors, without undue reservation.

## Ethics Statement

Ethical review and approval was not required for the study on human participants in accordance with the local legislation and institutional requirements. The patients/participants provided their written informed consent to participate in this study.

## Author Contributions

BL planned Study 1. BL, PW, and SS planned Study 2. BL prepared the materials and supervised data collection for both studies. BL and SS analyzed the data. BL, PW, and SS wrote the manuscript. All authors contributed to the article and approved the submitted version.

## Conflict of Interest

The authors declare that the research was conducted in the absence of any commercial or financial relationships that could be construed as a potential conflict of interest.
